# Case Report: Therapeutic plasma exchange for severe unconjugated hyperbilirubinemia with bilirubin-induced neurologic dysfunction in an adolescent with Crigler–Najjar syndrome type II

**DOI:** 10.3389/fped.2026.1845433

**Published:** 2026-08-17

**Authors:** Mohanad Jaber, Ammir Abuzahra, Ayah Kamel Karajat, Bara Abu Shama'a, Lama K. Hunyhen, Lara Baradia, Loai Muhtasib, Ashraf Alzughayyar, Hasan I. H. Hroob, Majed Dwaik, Yousef Al-Takrori

**Affiliations:** 1 Faculty of Medicine, Palestine Polytechnic University, Hebron, Palestine; 2 College of Medicine, Hebron University, Hebron, Palestine; 3 Medical Intensive Care Unit, Al-Ahli Hospital, Hebron, Palestine

**Keywords:** bilirubin encephalopathy, case report, Crigler–Najjar syndrome, therapeutic plasma exchange, unconjugated hyperbilirubinemia

## Abstract

**Background:**

Crigler–Najjar syndrome type II (CNS-II) is a rare inherited disorder of bilirubin conjugation that typically responds to phenobarbital and is less frequently complicated by bilirubin neurotoxicity than CNS-I. During physiologic stress, unconjugated bilirubin may acutely rise, potentially increasing unbound bilirubin and neurologic risk.

**Case presentation:**

A 15-year-old male with a known history of CNS-II presented with two weeks of progressive abnormal movements and worsening jaundice, followed by decreased consciousness and incoherent speech. Initial testing demonstrated severe isolated hyperbilirubinemia with minimal direct bilirubin fraction and no laboratory evidence of hepatocellular injury or synthetic dysfunction. He was transferred to the medical Intensive Care Unit with suspected bilirubin-induced neurologic dysfunction.

**Intervention:**

Given ongoing neurologic deterioration and severe hyperbilirubinemia despite supportive management, therapeutic plasma exchange (TPE) was initiated on the day of ICU admission and performed daily for seven sessions via central venous access with fresh frozen plasma used as the replacement fluid.

**Outcome:**

Total bilirubin declined from 35.0 mg/dL on admission (16-Feb-2025) to 28.8 mg/dL by session 2 (17-Feb-2025),to 27.4 mg/dL by session 3 (18-Feb-2025), to 24 mg/dL by sessions 4-5 (19-20-Feb-2025),to 19.5 mg/dL by sessions 6-7(21-22-Feb-2025) and approximately 18.8 mg/dL after seven sessions (23-Feb-2025), accompanied by clinical improvement in alertness and abnormal movements. Brain MRI obtained after TPE sessions demonstrate no Acute Bilirubin Encephalopathy-pattern or basal ganglia involvement.

**Conclusion:**

This case supports consideration of TPE as a rescue strategy for severe unconjugated hyperbilirubinemia with neurologic dysfunction in older children with CNS-II when conventional measures are insufficient or impractical, while underscoring the need for standardized reporting of TPE protocols and longer-term neurodevelopmental outcomes.

## Introduction

Crigler–Najjar syndrome is a rare inherited disorder caused by impaired bilirubin glucuronidation due to *UGT1A1* deficiency, resulting in persistent unconjugated hyperbilirubinemia (1). CNS type II (CNS-II) generally follows a milder clinical course than CNS type I; however, transient exacerbations of hyperbilirubinemia may occur during physiologic stress and can increase the risk of bilirubin-induced neurotoxicity, particularly when bilirubin–albumin binding capacity is exceeded or binding affinity is reduced (2). Therapeutic options for bilirubin control in CNS-II include phenobarbital induction, phototherapy (with decreasing practicality and efficacy in older patients), and extracorporeal modalities such as therapeutic plasma exchange (TPE) or plasmapheresis in severe or refractory crises (1). Although case-based literature suggests that exchange plasmapheresis can rapidly reduce plasma bilirubin levels and may be associated with clinical improvement in CNS-related neurologic deterioration, the available evidence remains limited, heterogeneous, and largely restricted to case reports and small case series, particularly in severe form of Crigler–Najjar syndrome (3). Here, we report an adolescent with genetically confirmed CNS-II harboring a homozygous *UGT1A1* variant (p.W461R) who developed severe unconjugated hyperbilirubinemia with neurologic dysfunction and was successfully treated with a short course of therapeutic plasma exchange, emphasizing bilirubin kinetics, diagnostic reasoning, and protocol-level reporting in accordance with CARE guidelines.

## Case description

A 15-year-old male with known Crigler–Najjar syndrome type II; as he was diagnosed based on genetic testing, which revealed a p.W461R (homozygous) mutation in exon 5 of the *UGT1A1* gene. Presented after two weeks of progressive abnormal movements (described as choreoathetoid/posturing episodes) and worsening jaundice, followed by several days of decreased level of consciousness, hypoactivity, and incomprehensible speech. He also had diarrhea shortly before the Intensive Care Unit (ICU) transfer. There was a family history of similarly affected siblings and parental consanguinity.

On initial outside evaluation, he appeared ill and deeply jaundiced without meningeal signs. Laboratory evaluation documented total bilirubin 40 mg/dL with direct bilirubin 0.8 mg/dL, consistent with marked predominantly unconjugated hyperbilirubinemia.

He was transferred to the medical ICU on 16-Feb-2025 for suspected bilirubin-induced neurologic dysfunction “Acute Bilirubin Encephalopathy”.

Vital signs were stable on room air. He was described as hypoactive with episodes of agitation; he was not in respiratory distress. Neurologic status was retrospectively approximated using the Glasgow Coma Scale (GCS), given the lack of consistently documented standardized neurologic scoring. At ICU admission, the patient had a retrospectively estimated GCS of 11 (E3V3M5), reflecting decreased level of consciousness and incomprehensible speech. During the period of maximal neurologic impairment, GCS was approximately 10–11, with intermittent agitation and reduced responsiveness. Following initiation of therapeutic plasma exchange, neurologic status improved progressively, with GCS increasing to 14 (E4V4M6) by the end of the ICU course. No focal motor deficit was documented; abnormal movements were described intermittently. A formal prospective neurologic severity score (e.g., Glasgow Coma Scale or neonatal-focused BIND/BIND-M tools) was not available in the reviewed medical records. Because BIND scores were developed and validated for neonates/infants, we instead provide (a) structured qualitative neurologic descriptors and (b) objective bilirubin kinetics as the primary measurable correlate.

We acknowledge that GCS is an imperfect surrogate for bilirubin-induced neurologic dysfunction (BIND), as it primarily measures level of consciousness rather than the characteristic extrapyramidal, auditory, or movement-related manifestations associated with bilirubin neurotoxicity. In addition, GCS has not been validated specifically for monitoring BIND severity in adolescent patients. However, neonatal-focused tools such as BIND or BIND-M were not applicable because these instruments were developed and validated primarily in neonates and young infants. Consequently, neurologic assessment in this case relied on a combination of retrospective GCS estimation, structured qualitative neurologic descriptors, and objective bilirubin response kinetics to document clinical progression and recovery.

Serial chemistry supported isolated hyperbilirubinemia with minimal direct bilirubin elevation and no evidence of hepatocellular injury (AST/ALT within reference) or significant coagulopathy (INR ∼1.24). CBC values showed no anemia and platelets within reference ranges; mild leukocytosis was intermittently present (Table 1).

**Table 1:** Serial laboratory parameters obtained during therapeutic plasma exchange (TPE) sessions.

Lab values	Hemoglobin	WBCs	Platelets	Aspartate Aminotransferase (AST/SGOT)	Alanine Aminotransferase (ALT/SGPT)	Alkaline Phosphatase	Fibrinogen	INR	LACTIC ACID	Ammonia
Reference range	12–16 g/dL	5.0–10.0 × 10^3^/µL	150–400 × 10^3^/µL	0–37 U/L	0–45 U/L	115–460 U/L	150–350 mg/dL	0.8–1.2	5.7–22 mg/dL (plasma)	31–123 UG/dL
16-Feb	14.46	8.682	344.9	29.0	24.0	193.0	417	1.17	14.0	102.0
17-Feb	14.33	16.13	287.2	17.0	7.0	112.0	229	-		
18-Feb	14.47	12.8	249.3	21.0	3.0	85.0	245	-		
19-Feb	15.48	13.44	263.5	26.0	14.0	73.0	-	1.24		
20-Feb	14.47	9.267	213.5	25.0	16.0	78.0	-	-		
21-Feb	13.37	10.07	282.1	25.0	16.5	75.0	197	1.40		
22-Feb	12.84	11.33	254.2	19.0	19.0	68.0	176	1.3		

INR, international normalized ratio; SGOT, serum glutamic-oxaloacetic transaminase (AST); SGPT, serum glutamic-pyruvic transaminase (ALT); WBCs, white blood cells; TPE, therapeutic plasma exchange.

The table demonstrates trends in liver function markers, coagulation profile, ammonia level, hematologic indices, and metabolic parameters throughout the treatment course. Liver enzyme levels (AST, ALT, and alkaline phosphatase) remained within the reference range throughout consecutive TPE sessions, while fibrinogen levels demonstrated progressive improvement.

Abdominal ultrasound showed mild hepatosplenomegaly was described (liver 15.7 cm MCL; spleen 12.8 cm) with gallbladder sludge but no biliary dilation and no free fluid.

**Figure 1:**
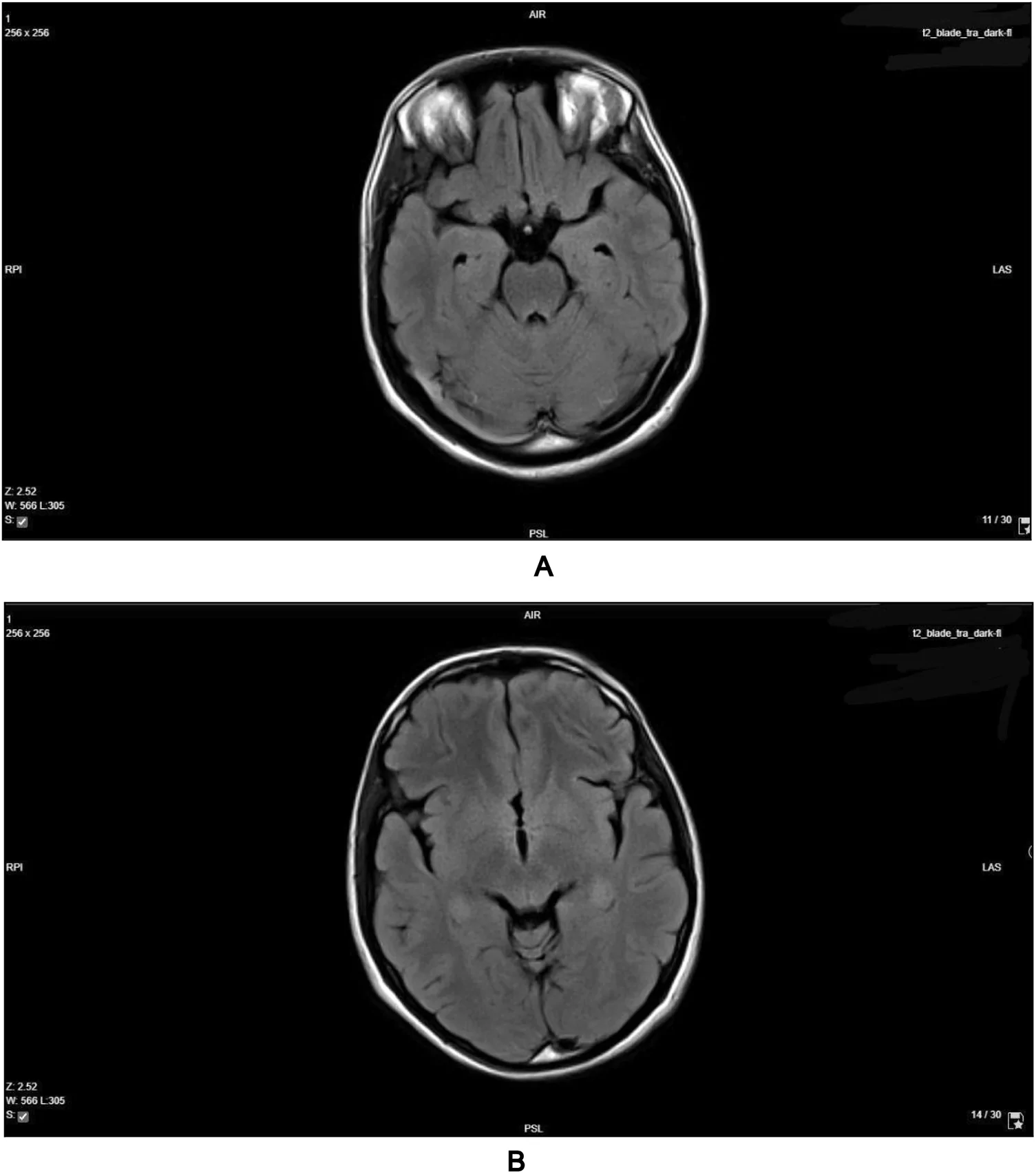
**(A,B)**. Brain MRI obtained after TPE sessions (axial FLAIR) demonstrating no Acute Bilirubin Encephalopathy-pattern or basal ganglia involvement. **(A,B)** Axial FLAIR images at different levels demonstrate no definite abnormal signal intensity within the basal ganglia or other findings suggestive of bilirubin-induced neurologic dysfunction.

Post-TPE sessions Brain MRI Axial FLAIR images demonstrated no Acute Bilirubin Encephalopathy-pattern or basal ganglia involvement; representative images are shown in (Figures 1A,B).

Differential diagnoses considered and rationale included bilirubin-induced neurologic dysfunction in the context of severe unconjugated hyperbilirubinemia and a compatible movement disorder phenotype, which was considered the most likely diagnosis. Seizure disorder or encephalitis were considered less likely given the patient's systemic stability and lack of supporting clinical data, although cerebrospinal fluid testing was not available in the reviewed medical records. Hepatic encephalopathy secondary to liver failure was also considered less likely due to preserved transaminase levels and normal INR values. Drug or toxin exposure was considered unlikely because no supportive history was available. Metabolic movement disorders were likewise considered less likely in the context of the known central nervous system involvement and bilirubin crisis physiology.

### Therapeutic intervention

#### Initial management (outside hospital and on arrival)

Phenobarbital therapy was continued at a dose of 120 mg/day (approximately 2.7 mg/kg/day) as part of baseline management for Crigler–Najjar syndrome type II. Adherence prior to admission could not be fully confirmed, which may have contributed to the marked elevation in bilirubin levels at presentation. Supportive care included Intravenous hydration with normal saline at 100 mL/hour and close monitoring of fluid balance through assessment of urine output and oral intake.

#### Apheresis decision-making

Given severe unconjugated hyperbilirubinemia with neurologic dysfunction and concern for bilirubin neurotoxicity (including risks mediated by unbound bilirubin), an extracorporeal bilirubin-reduction strategy was selected. Literature supports the physiologic rationale that plasma exchange removes albumin-bound bilirubin and can rapidly reduce plasma bilirubin in CNS crises.

#### Therapeutic plasma exchange (TPE) course

Each therapeutic plasma exchange session involved approximately 1–1.5 times the estimated plasma volume, performed using citrate-based anticoagulation with routine calcium supplementation to prevent hypocalcemia. Replacement fluid consisted primarily of fresh frozen plasma, as documented in blood bank records. Each session lasted approximately 2–3 h and was performed via central venous access under continuous hemodynamic monitoring. No procedure-related complications were observed. - Start date: 16-Feb-2025 (hospital day 1). - Frequency: daily. - Total sessions: 7. - Access: central venous catheter placed on admission day (site not specified in the reviewed medical records).

- Replacement fluid: donor fresh frozen plasma is documented in blood bank requisitions; albumin was also used as partial replacement and calcium supplementation protocol done as citrate was used.
- Anticoagulation/exchanged plasma volume/session duration/stopping rules: Therapeutic plasma exchange was performed using a B. Braun apheresis system. Fresh frozen plasma (FFP) was used as the replacement fluid. The procedure was conducted with zero net fluid removal. Based on the patient's body weight of 45 kg, the estimated plasma volume was approximately 1800 mL (calculated at approximately 40 mL/kg), and approximately 1–1.5 times the estimated plasma volume was exchanged according to institutional protocol. The maximum transmembrane pressure (TMP) recorded during the procedure was 160 mmHg.

#### Complications

No major TPE complications were documented in the available chart images (hypocalcemia symptoms, bleeding, line infection, or hemodynamic instability not recorded).

### Follow-up and outcomes

#### Bilirubin kinetics (primary quantitative outcome)

**Figure 2:**
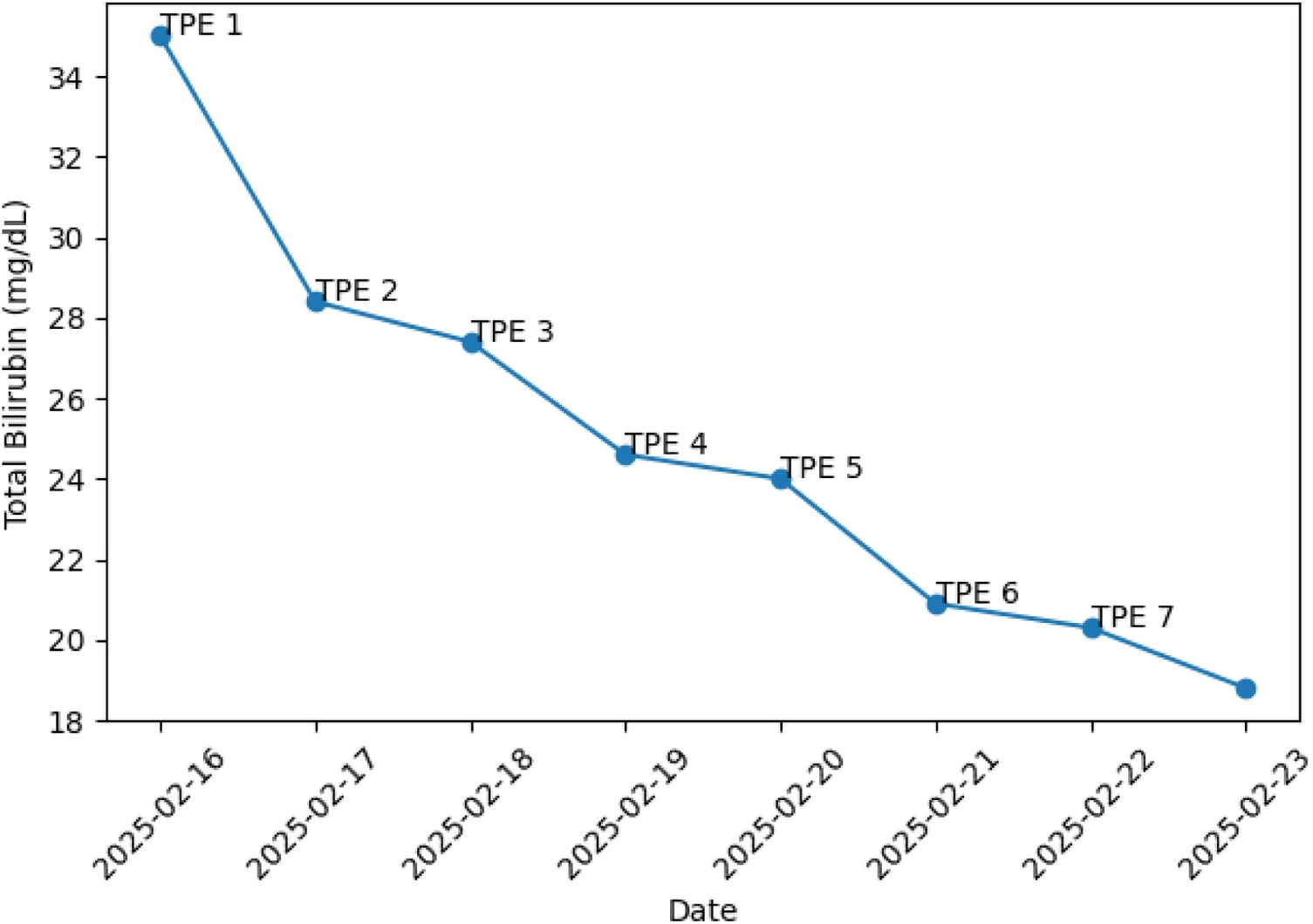
Serial total bilirubin trend during ICU course and therapeutic plasma exchange. Total bilirubin (mg/dL) from 16-Feb-2025 to 23-Feb-2025 demonstrates a progressive decline during a seven-session daily therapeutic plasma exchange course, from 35.0 mg/dL to approximately 18.8 mg/dL. Markers indicate individual TPE sessions; values represent pre-session measurements.

Total bilirubin decreased from 35.0 mg/dL (16-Feb) to 28.4 mg/dL (17-Feb), 27.4 mg/dL (18-Feb), and 24.6 mg/dL by sessions 4–5 (19–20 Feb), reaching approximately 18.8 mg/dL after seven sessions (23-Feb) (Figure 2). This represents an overall reduction of ∼16.2 mg/dL (≈46% relative decrease). Neurotoxicity in bilirubin-induced neurologic dysfunction is primarily mediated by unbound (free) bilirubin rather than total bilirubin alone, supporting the use of extracorporeal therapies such as plasma exchange to reduce circulating bilirubin and its neurotoxic fraction. Nevertheless, total bilirubin and bilirubin–albumin metrics remain clinically useful surrogate markers in the broader bilirubin encephalopathy literature.

#### Clinical course

Neurologic status improved in parallel with bilirubin reduction, with documented improvement in alertness and reduced abnormal movement episodes. He remained hemodynamically stable and oxygenated on room air in a later ICU course.

### Discharge and long-term follow-up

The patient showed sustained clinical and biochemical improvement following completion of seven sessions of therapeutic plasma exchange. By hospital day 8, he was alert, with marked reduction in abnormal movements and improved interaction. Total bilirubin decreased to approximately 18.8 mg/dL. He remained hemodynamically stable, required no respiratory support, and tolerated oral intake prior to discharge. No procedure-related complications were observed.

The patient was discharged on continued phenobarbital therapy with strong emphasis on adherence. Caregivers were counseled regarding early recognition of neurologic symptoms and the need for prompt medical evaluation during intercurrent illness.

Follow-up was arranged for regular monitoring of bilirubin levels and neurologic status. The patient was followed regularly in the outpatient clinic on a monthly basis. Persistent neurologic improvement was noted during follow-up, and he remained clinically stable without recurrence of severe neurologic symptoms. The clinical course, interventions, and bilirubin trends are summarized in Table 2.

**Table 2:** Timeline of key events, interventions, and bilirubin trend (mg/dL).

Date (2025)	Hospital day	Key clinical events	Intervention	Total bilirubin (mg/dL)	Direct bilirubin (mg/dL)
14-Feb	—	Referral from an external Emergency Department for abnormal movements + jaundice	Supportive care	40.0	0.8
16-Feb	1	ICU transfer for suspected bilirubin-induced neurologic dysfunction	TPE session 1	35.0	—
17-Feb	2	Ongoing neurologic dysfunction	TPE session 2	28.8	0.5
18-Feb	3	Clinical stabilization	TPE session 3	27.4	0.5
19–20 Feb	4–5	Improved alertness	TPE sessions 4–5	24.0	0.5
21–22 Feb	6–7	Continued improvement	TPE sessions 6–7	∼19.5	0.4
23-Feb	8	Post-TPE monitoring	Supportive	∼18.8	0.3

TPE, therapeutic plasma exchange.

Chronological summary of clinical course, therapeutic plasma exchange (TPE), and bilirubin levels during hospitalization.

## Discussion

This case highlights the potential for severe bilirubin crises and neurologic dysfunction in patients with Crigler–Najjar syndrome type II (CNS-II), a condition traditionally considered less severe than type I. Although CNS-II is typically responsive to phenobarbital and associated with a lower risk of Acute Bilirubin Encephalopathy, significant neurotoxicity can still occur, particularly during periods of physiologic stress. The risk of neurologic injury is not determined solely by total bilirubin levels but is strongly influenced by bilirubin–albumin binding dynamics and the fraction of unbound (free) bilirubin, which is the primary mediator of neurotoxicity (1).

Therapeutic plasma exchange (TPE) in this case was associated with a substantial and progressive reduction in total bilirubin levels, accompanied by parallel clinical improvement. The mechanistic rationale for TPE is well established: bilirubin is predominantly albumin-bound in circulation, and plasma exchange facilitates removal of bilirubin-bound albumin while replacing it with fresh plasma, thereby reducing both total and free bilirubin fractions (4). Since free (unbound) bilirubin is the main neurotoxic fraction capable of crossing the blood–brain barrier, its reduction may help prevent or reverse bilirubin-induced neurologic dysfunction (5–7). Pediatric patients may require multiple TPE sessions, each lasting approximately 90 min to 2 h. Adequate hydration and supportive care are important, as transient side effects such as dizziness, fatigue, and hypotension may occur during treatment (6, 8, 9). Previous reports have demonstrated similar effects, including marked bilirubin reduction and clinical improvement in patients with Crigler–Najjar syndrome type I following plasmapheresis. Our case extends this evidence to an adolescent patient with CNS-II, supporting the role of TPE as a viable therapeutic option in severe hyperbilirubinemic crises beyond the neonatal period, while acknowledging the limited and non-systematic nature of the available literature (10). A summary of selected published cases is provided in Table 3.

**Table 3:** Published evidence for plasma exchange/plasmapheresis in crigler–najjar crises (selected cases).

Ref	CNS type/age	Clinical context	Intervention	Quantitative bilirubin response	Reported outcome
1 (3)	Type I; 18 years	Neurologic signs; very high bilirubin	Exchange plasmapheresis	41 → 6 mg/dL	Apparent clinical improvement
2 (9)	Type I; 6 years	Acute severe UCHB despite intensified phototherapy	Plasmapheresis ×2	Reported marked decline (mg/dL)	Rapid biochemical improvement
3 (5)	Type I; 18 years	Recurrent CNS symptoms with rising free bilirubin	Repeated plasma exchanges	Long-term maintenance reported	Symptom control reported
4 (this case)	Type II phenotype; 15 years	UCHB crisis + neurologic dysfunction	TPE daily × 7	35.0 → ∼18.8 mg/dL	Clinical improvement with bilirubin decline

UCHB, unconjugated hyperbilirubinemia.

Selected reported cases summarizing patient characteristics, interventions, bilirubin response, and outcomes, including the present case.

Therapeutic plasma exchange (TPE) works by removing the patient's plasma and replacing it with donor plasma or albumin-containing fluids, thereby eliminating circulating pathogenic or toxic substances from the bloodstream. In hyperbilirubinemic conditions such as Crigler-Najjar syndrome, bilirubin is predominantly bound to albumin in circulation. TPE removes bilirubin-bound albumin and replaces it with fresh plasma containing unbound albumin, which increases bilirubin-binding capacity and reduces both total and free bilirubin levels.

Neuroimaging findings in this case were unremarkable. Brain MRI performed after the TPE sessions demonstrated no acute bilirubin encephalopathy pattern or basal ganglia involvement (11). A normal post-treatment brain MRI does not exclude the possibility of prior radiologic abnormalities, as imaging findings may have resolved following therapy. Pre-treatment MRI was unavailable because the patient's care involved multiple centers with limited communication and incomplete transfer of imaging data before treatment initiation. Although characteristic MRI abnormalities have been described in acute bilirubin encephalopathy, imaging findings may vary depending on the timing of image acquisition and the MRI sequences used. Therefore, the absence of radiologic abnormalities does not exclude bilirubin-induced neurologic dysfunction, emphasizing the importance of correlating clinical presentation and biochemical findings when evaluating suspected bilirubin neurotoxicity (12).

The choice of therapeutic plasma exchange over exchange transfusion reflects important clinical considerations. While exchange transfusion is a well-established intervention in neonatal hyperbilirubinemia, its application in older children and adolescents is less clearly defined. In this population, factors such as larger circulating blood volume, procedural complexity, and institutional expertise may limit its feasibility (13). In contrast, TPE offers a targeted extracorporeal approach for removing albumin-bound toxins and has been successfully utilized in similar contexts, making it a rational and practical alternative in selected cases (4).

## Conclusion

In an adolescent with Crigler–Najjar syndrome type II and severe unconjugated hyperbilirubinemia with neurologic dysfunction, a short daily course of therapeutic plasma exchange was associated with a substantial bilirubin decline and concurrent clinical improvement. This report supports consideration of TPE as a rescue strategy in severe CNS crises when conventional measures are insufficient or impractical, while emphasizing the need for standardized protocol reporting and long-term neurologic outcome assessment.

## Data Availability

The original contributions presented in the study are included in the article/Supplementary Material, further inquiries can be directed to the corresponding author.
